# Regional, demographic, and temporal trends in myeloid leukemia mortality in the United States (1999-2022): a comprehensive analysis using CDC WONDER

**DOI:** 10.3389/fonc.2025.1560797

**Published:** 2025-07-11

**Authors:** Jenna Lehn, Hannah Fleming, Taylor Billion, Mohsin Mirza

**Affiliations:** ^1^ Creighton University School of Medicine, Omaha, NE, United States; ^2^ Creighton University Department of Internal Medicine, Omaha, NE, United States

**Keywords:** mortality, myeloid leukemia, database, region, demographic, trend

## Abstract

Myeloid Leukemias (ML) are neoplastic disorders characterized by the abnormal production of myeloid blood cells and disrupted hematopoiesis. Limited research exists on regional and demographic trends in ML mortality. This study investigates ML mortality patterns in the U.S. from 1999 to 2022, focusing on geographic and demographic disparities using age-adjusted mortality rates (AAMR) and average annual percent change (AAPC). Data were obtained from the CDC WONDER database, with AAMRs calculated per 100,000 people and stratified by region, state, urbanicity, sex, and race. AAPCs were computed using the National Cancer Institute’s (NCI) Joinpoint Regression Program (Joinpoint V 4.9.0.0, NCI). Between 1999 and 2022, there were 299,221 ML-related deaths nationwide. While most U.S. regions showed a non-significant downward trend in mortality, the Midwest demonstrated a non-significant upward trend and consistently higher AAMRs. States with the highest AAMRs included Iowa, Kansas, Minnesota, and the Dakotas—predominantly rural states. Rural areas overall had higher AAMRs than urban areas. Males and White individuals had the highest mortality across all regions, with the Midwest showing the highest AAMRs for both sexes. Although ML mortality declined significantly from 1999 to 2007, it showed a non-significant increase from 2007 to 2022, despite therapeutic advancements. Persistent disparities—particularly among rural Midwestern populations, White patients, and males—highlight the need for targeted interventions and further research to address these geographic and demographic inequities.

## Introduction

Myeloid Leukemias (ML) are neoplastic disorders that cause the production of abnormal myeloid blood cells and irregular hematopoiesis (1). Primarily divided into two major types, acute myeloid leukemia (AML) is more common than chronic myeloid leukemia (CML) (1). There are estimated to be 29,310 new cases of ML diagnosed in 2023, and 12,620 expected deaths in the U.S (2). This accounts for 15-20% of leukemias in the US (3). These clonal malignancies have had a relatively unchanged treatment regimen for the past 40 years, which consists of a combination of anthracyclines (3).

Previous studies have investigated various mortality trends in ML in the US (4, 5). However, these studies lacked a comprehensive analysis of geographic and regional variation. This study uses the Center for Disease Control and Prevention (CDC) WONDER database to comprehensively investigate differences in the age-adjusted mortality rates (AAMRs) for myeloid leukemias based on region, state, urban/rural residence, gender, and race in the U.S. from 1999-2022. With different primary affected age groups and clinical presentation, AML and CML diverge in epidemiological factors. Genetic and biological differences between AML and CML require them to have tailored treatment and management plans. However, when investigating overarching sociodemographic trends, we believe the combination of AML and CML into the extended category of ML provides more insight into cancer burden. This would contribute value in understanding the broader epidemiological trends and public health implications of ML. While there has been significant study of AML and CML, overarching ML trends have been studied and should be further explored in the literature (6). The (CDC) WONDER database allows the study of all subtypes of ML, and it is for these reasons our overall aim is to understand the trends and overall burden of ML on the healthcare system, as opposed to the individual subtypes of leukemia. By analyzing these trends, we aim to advocate for further database sharing and research on ML with the hope of enabling providers and healthcare officials to advance management plans and policies to address MLL disparities.

## Methods

ML mortality was analyzed from 1999–2022 in the U.S. using the Centers for Disease Control and Prevention Wide-Ranging Online Data for Epidemiologic Research (CDC WONDER) database (7). CDC Wonder death certificate records and the Multiple Cause-of-Death Public Use Record were used to determine ML-related causes of death. Prior studies have used the CDC WONDER database to analyze ML mortality and its trends (5). The International Classification of Diseases, 10th Revision, Clinical Modification code C92 in patients one year or older was used to analyze ML-related mortality. The prevalence of ML in patients under one year of age was too low to include in the dataset. This study did not require Institutional Review Board approval because CDC WONDER is a publicly available database that contains anonymous data.

Data was extracted from 1999–2022 for ML-related deaths and population sizes. Demographic and regional group data, including gender, race/ethnicity, urban-rural classification, region, and states, were extracted. Racial/ethnicity groups were defined as White, Black, Asian/Pacific Islander, and Hispanic/Latino. Urban classifications were divided into large metropolitan areas (population ≥1 million), medium/small metropolitan areas (population 50,000 to 999,999), while rural had their classification (population <50,000), as per the 2013 United States census classification in the National Center for Health Statistics Urban-Rural Classification Scheme (8). Regions were separated into Northeast, Midwest, South, and West according to the Census Bureau definitions (9).

ML-related AAMRs were standardized using the 2000 U.S. standard population (10). The Joinpoint Regression Program (Joinpoint version 4.9.0.0 available from National Cancer Institute, Bethesda, Maryland) was used to determine mortality trends from 1999-2022 (11). Annual percent change (APC) and average annual percent change (AAPC) with 95% confidence intervals (CIs) for the AAMRs were calculated for the line segments linking a Joinpoint using the Monte Carlo permutation test. If the slope describing the change in mortality over time significantly differed from zero using a 2-tailed t-test, APC and AAPCs were considered increasing or decreasing. Statistical significance was set at p ≤0.05. Asterisks were used to denote significance.

## Results

From 1999 to 2022, there were 299,221 deaths due to myeloid leukemia in the United States.

**Figure 1:**
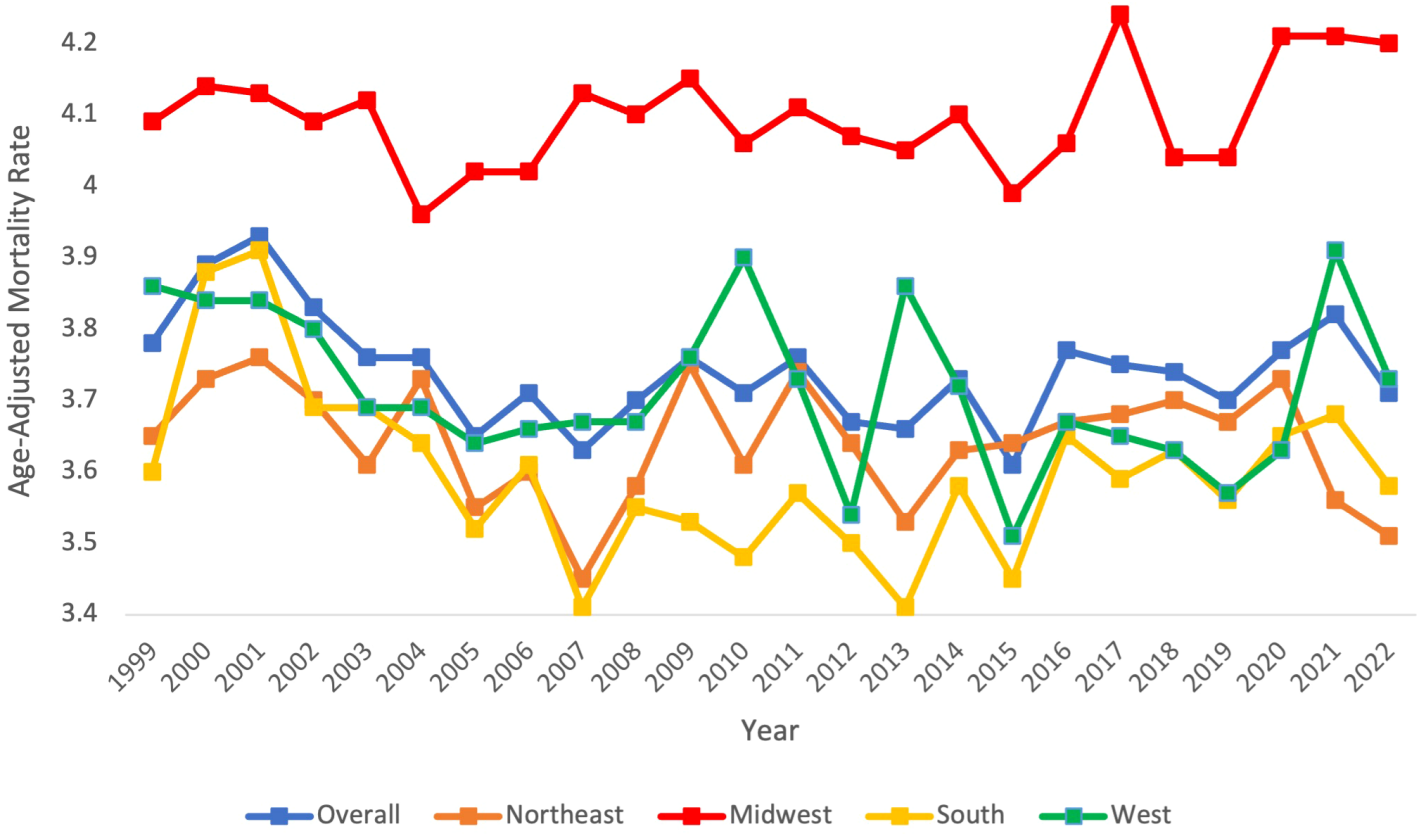
Myeloid Leukemia cancer age-adjusted mortality rate per 100,000 people; overall and stratified by region, 1999-2022.

**Figure 2:**
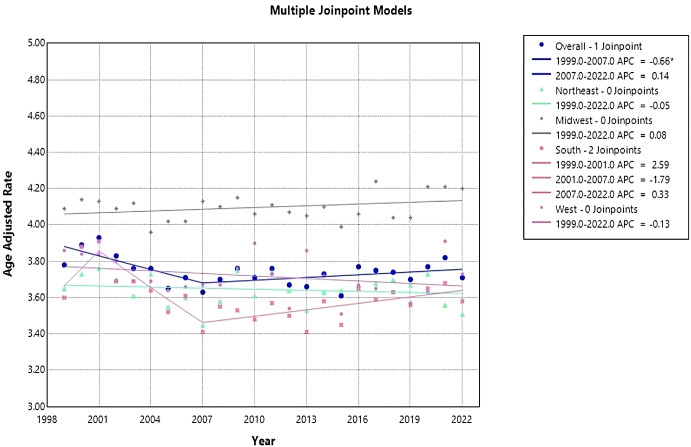
Joinpoint model of Myeloid Leukemia cancer-related AAMR per 100,000 people; overall and stratified by region, 1999-2022 (*indicates the APC is statistically significant).

Overall age-adjusted mortality rates (AAMR) decreased during this period from 3.78 (95% CI 3.7 to 3.85) in 1999 to 3.71 (95% CI 3.65 to 3.77) in 2022 (**Figure 1**, **Table 1**). The annual percentage change (APC) showed a significant decrease at -0.66 (95% CI -2.80 to -0.07)* from 1999-2007, which increased to 0.14 (95% CI -0.06 to 1.15) from 2007-2022 (**Figure 2**). Overall, the AAMR was the lowest in 2015 and increased from 3.16 (95% CI 3.54 to 3.67) to 3.71 (95% CI 3.65 to 3.77) by 2022 (**Figure 1**).

**Table 1:** Myeloid Leukemia cancer age-adjusted mortality rate per 100,000 people; overall and stratified by region, 1999-2022.

Year	Overall	Northeast	Midwest	South	West
1999	3.78	3.65	4.09	3.60	3.86
2000	3.89	3.73	4.14	3.88	3.84
2001	3.93	3.76	4.13	3.91	3.84
2002	3.83	3.70	4.09	3.69	3.80
2003	3.76	3.61	4.12	3.69	3.69
2004	3.76	3.73	3.96	3.64	3.69
2005	3.65	3.55	4.02	3.52	3.64
2006	3.71	3.60	4.02	3.61	3.66
2007	3.63	3.45	4.13	3.41	3.67
2008	3.70	3.58	4.10	3.55	3.67
2009	3.76	3.75	4.15	3.53	3.76
2010	3.71	3.61	4.06	3.48	3.90
2011	3.76	3.74	4.11	3.57	3.73
2012	3.67	3.64	4.07	3.5	3.54
2013	3.66	3.53	4.05	3.41	3.86
2014	3.73	3.63	4.10	3.58	3.72
2015	3.61	3.64	3.99	3.45	3.51
2016	3.77	3.67	4.06	3.65	3.67
2017	3.75	3.68	4.24	3.59	3.65
2018	3.74	3.70	4.04	3.63	3.63
2019	3.70	3.67	4.04	3.56	3.57
2020	3.77	3.73	4.21	3.65	3.63
2021	3.82	3.56	4.21	3.68	3.91
2022	3.71	3.51	4.20	3.58	3.73

### Census regions

Each census region apart from the Midwest saw a decrease in AAMR from 1999-2022, with the Northeast having the largest reduction in AAMR from 3.65 (95% CI 3.49 to 3.81) in 1999 to 3.51 (95% CI 3.37 to 3.64) in 2022 (**Table 1**, **Figure 1**). The Northeast had a consistent AAPC and APC of 0.05 (95% CI -0.22 to 0.13) (**Figure 2**). The Midwest had an increase in AAMR from 4.09 (95% CI 3.93 to 4.24) in 1999 to 4.2 (95% CI 4.07 to 4.34) in 2022, with a consistent AAPC and APC of 0.08 (95% CI -0.06 to 0.22) (**Table 1**, **Figures 1**, **2**). The South had a decrease in AAMR from 3.6 (95% CI 3.48 to 3.72) in 1999 to 3.58 (95% CI 3.48 to 3.67) in 2022 (**Table 1**, **Figure 1**). From 1999-2001, the South had an APC of 2.6 (95% CI -1.41 to 7.28), in 2001–2007 the APC became -1.79 (95% CI -4.26 to 2.34), and from 2007–2022 the APC became 0.33 (95% CI -2.22 to 1.29) (**Figure 2**). The West had a decrease in AAMR from 3.86 (95% CI 3.70 to 4.02) in 1999 to 3.73 (95% CI 3.61 to 3.86) in 2022, with a consistent AAPC and APC of -0.13 (95% CI -0.32 to 0.09) (**Table 1**, **Figures 1**, **2**). From 1999-2022, the AAMR was highest in the Midwest at 4.24 (95% CI 4.09 to 4.38), followed by the South at 3.91 (95% CI 3.78 to 4.03), the West at 3.91 (95% CI 3.78 to 4.05), and the Northeast at 3.76 (95% CI 3.60 to 3.91) (**Table 1**, **Figure 1**). None of the regions saw a significant change in AAMR over time.

### State-level difference

The Average AAMR for the duration of the study period varied widely from 2.61 (95% CI 1.89 to 3.51) in Nevada to 5.38 (95% CI 4.07 to 6.99) in South Dakota (**Table 2**). States with an AAMR greater than the 90th percentile included Iowa, Kansas, Minnesota, North Dakota, and South Dakota. Conversely, states with an AAMR less than the 10th percentile included Connecticut, Hawaii, Nevada, New Mexico, and Wyoming (**Table 2**).

**Table 2:** Myeloid Leukemia cancer age-adjusted mortality rate per 100,000 people; stratified by state, 1999, 2019-2022.

State	1999	2019	2020	2022
Alabama	2.97	2.79	3.79	3.29
Alaska	*	3.48	2.95	*
Arizona	3.09	3.11	3.41	3.39
Arkansas	3.31	3.62	3.06	2.99
California	3.77	3.49	3.54	3.67
Colorado	4.29	3.59	3.70	4.11
Connecticut	2.86	4.09	3.62	3.43
Delaware	4.01	4.19	3.61	3.81
District of Columbia	*	*	3.63	3.66
Florida	3.91	3.56	3.74	3.64
Georgia	3.42	3.07	3.19	3.20
Hawaii	3.33	2.65	3.82	4.04
Idaho	3.70	3.77	4.35	4.00
Illinois	3.93	3.65	3.91	3.60
Indiana	3.75	4.07	3.66	4.23
Iowa	5.26	4.20	4.29	4.27
Kansas	3.90	4.60	5.24	4.05
Kentucky	3.60	4.34	4.89	4.22
Louisiana	4.43	3.28	3.36	3.32
Maine	2.99	4.17	3.97	3.85
Maryland	3.25	3.93	3.35	3.65
Massachusetts	3.64	3.46	3.56	3.29
Michigan	3.47	3.98	4.20	4.10
Minnesota	4.66	4.35	4.66	5.12
Mississippi	3.25	3.36	3.49	3.54
Missouri	4.42	3.46	3.47	3.64
Montana	4.95	3.47	3.16	3.47
Nebraska	4.25	4.80	4.27	3.66
Nevada	2.61	3.67	3.09	2.92
New Hampshire	3.85	4.14	3.42	3.69
New Jersey	3.54	3.24	3.71	3.28
New Mexico	3.33	2.94	3.03	2.73
New York	3.85	3.59	3.54	3.33
North Carolina	3.63	3.91	3.77	3.93
North Dakota	4.64	3.82	5.17	3.68
Ohio	3.90	4.09	4.43	4.32
Oklahoma	3.88	4.26	4.11	3.53
Oregon	3.56	4.26	4.18	4.66
Pennsylvania	3.68	3.94	4.06	3.99
Rhode Island	3.50	4.29	3.69	3.61
South Carolina	3.48	3.73	4.08	3.77
South Dakota	4.89	4.47	4.00	5.38
Tennessee	3.65	3.58	3.73	4.03
Texas	3.43	3.45	3.45	3.28
Utah	4.46	3.87	3.64	3.81
Vermont	3.41	3.58	4.96	3.39
Virginia	3.41	3.55	3.53	3.66
Washington	5.03	4.13	4.05	4.18
West Virginia	3.98	3.98	4.51	4.06
Wisconsin	4.58	4.46	4.92	5.01
Wyoming	4.91	4.82	4.47	2.86

(* indicates unreliable data).

**Figure 3:**
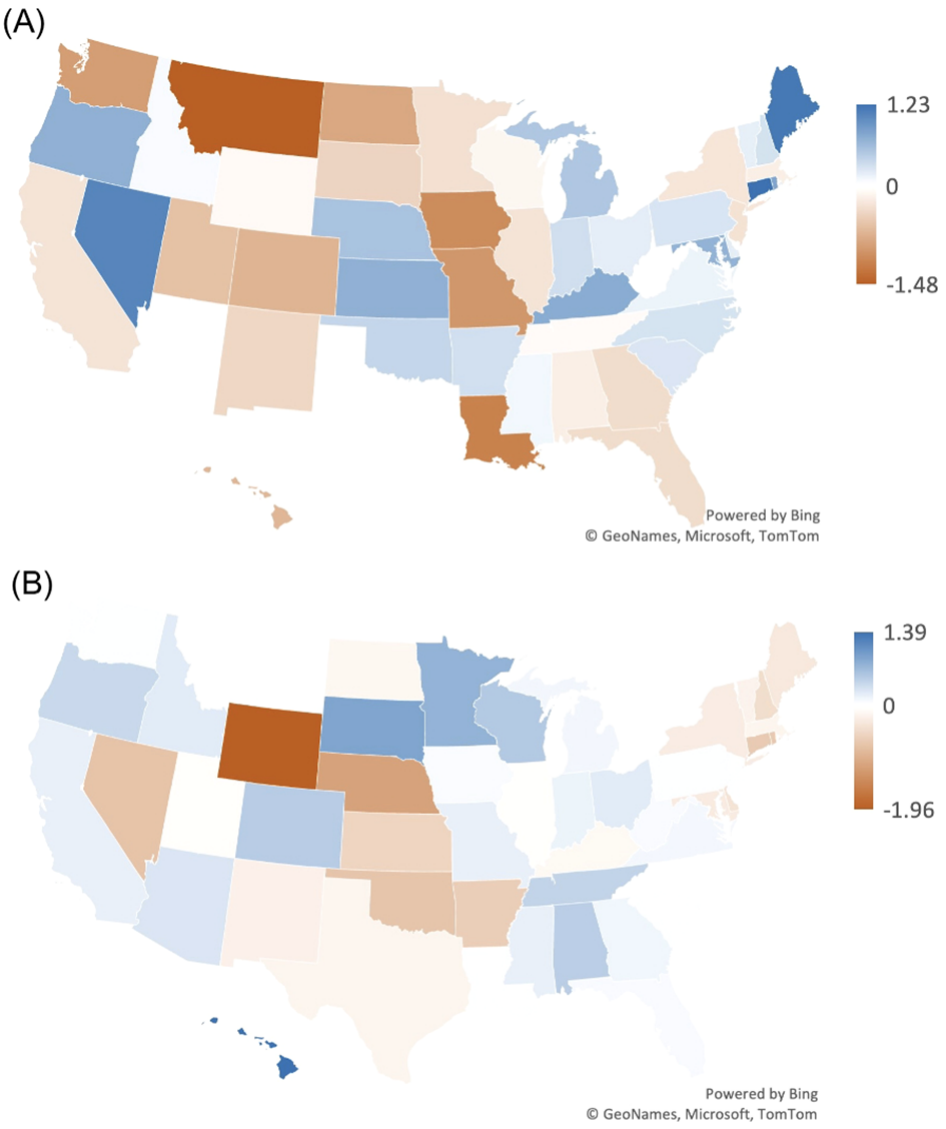
Map of Myeloid Leukemia cancer age-adjusted mortality rate per 100,000 people; stratified by state, Alaska and the District of Columbia had unreliable data. **(A)** 1999 vs 2019. **(B)** 2019 vs 2022.

Connecticut had the largest increase in AAMR from 1999 to 2019, followed by Maine and Nevada (**Table 2**, **Figure 3**). Conversely, Montana had the largest decrease, followed by Louisiana and Iowa (**Table 2**, **Figure 3**).

The states with the largest increase in AAMR from 2019–2022 included Hawaii, followed by South Dakota and Minnesota (**Table 2**, **Figure 3**). Conversely, the state with the largest decrease in AAMR from 2019–2022 included Wyoming, followed by Nebraska and Nevada (**Table 2**, **Figure 3**). Alaska and the District of Columbia had unreliable data.

### Rural v. urban

**Figure 4:**
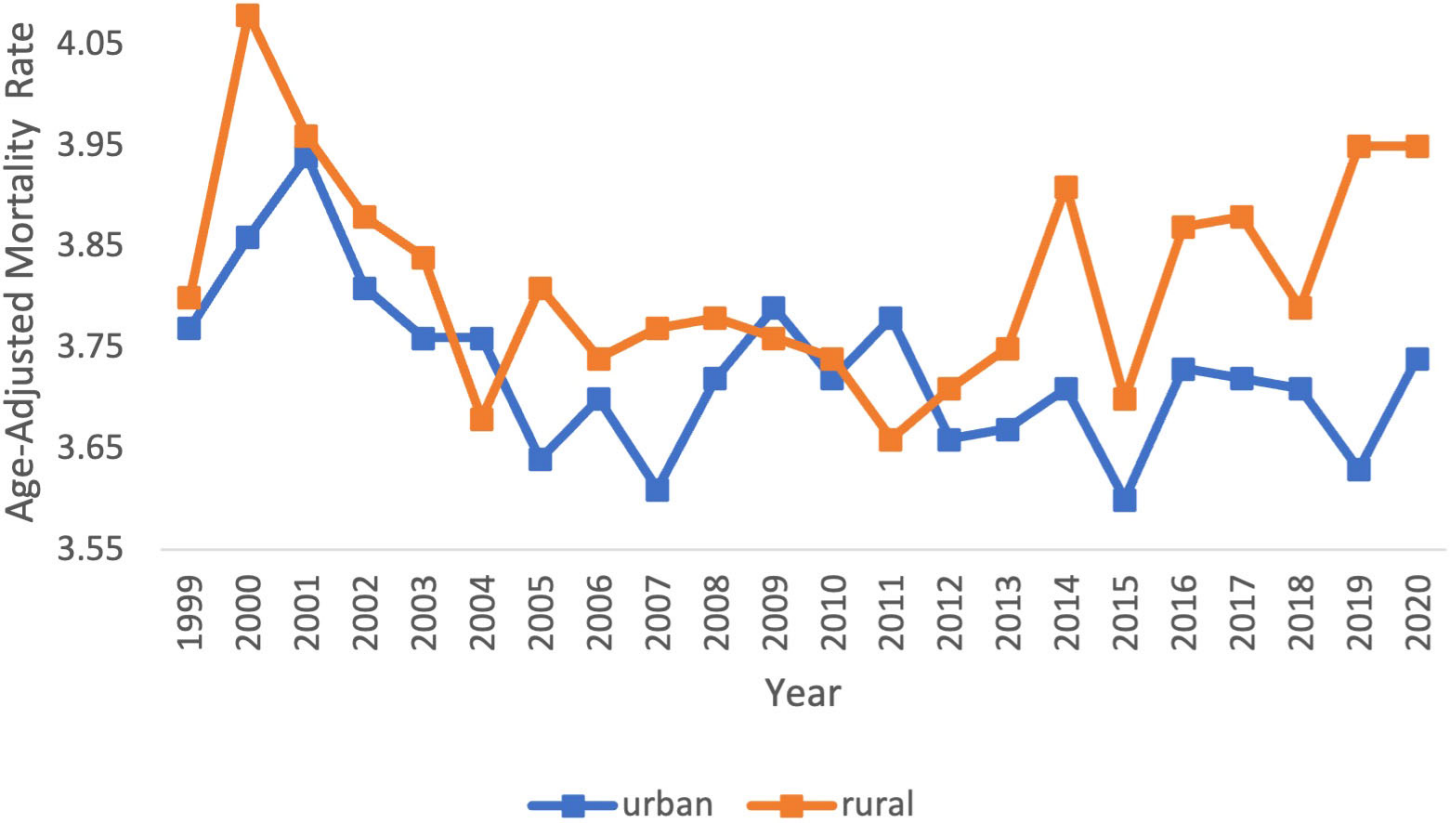
Myeloid Leukemia cancer age-adjusted mortality rate per 100,000 people; stratified by urban vs rural, 1999-2020.

**Figure 5:**
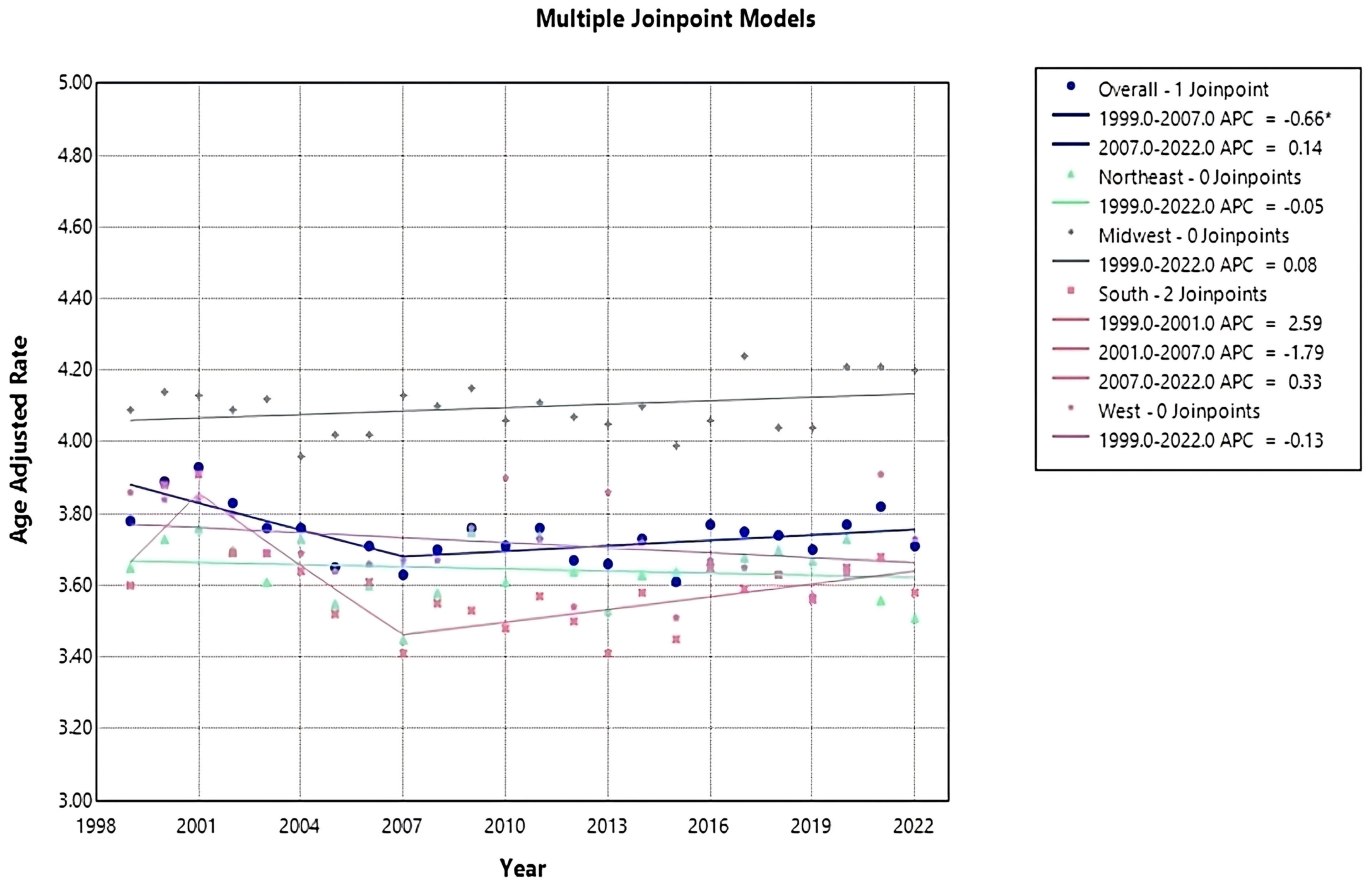
Joinpoint model of Myeloid Leukemia cancer-related AAMR per 100,000 people; overall and stratified by urban vs rural, 1999-2020 (*indicates the APC is statistically significant).

Across populated regions, AAMRs were persistently higher in rural areas as compared to small, medium, and large urban centers. Rural regions had a range of AAMRs from 3.80 (95% CI 3.63 to 3.98) in 1999 to 3.95 (95% CI 3.80 to 4.11) in 2020 (**Table 3**, **Figure 4**). In the rural regions from 1999-2011, there was a significant decrease in the APC of -0.51 (95% CI -1.23 to -0.19)* (**Figure 5**). Conversely, in the rural regions from 2011-2020, there was a significant increase in the APC of 0.73 (95% CI 0.27 to 1.81)* (**Figure 5**).

**Table 3:** Myeloid Leukemia cancer age-adjusted mortality rate per 100,000 people; stratified by urban vs rural, 1999-2020.

Year	Urban	Rural
1999	3.77	3.80
2000	3.86	4.08
2001	3.94	3.96
2002	3.81	3.88
2003	3.76	3.84
2004	3.76	3.68
2005	3.64	3.81
2006	3.70	3.74
2007	3.61	3.77
2008	3.72	3.78
2009	3.79	3.76
2010	3.72	3.74
2011	3.78	3.66
2012	3.66	3.71
2013	3.67	3.75
2014	3.71	3.91
2015	3.60	3.70
2016	3.73	3.87
2017	3.72	3.88
2018	3.71	3.79
2019	3.63	3.95
2020	3.74	3.95

Urban regions had a range of AAMRs from 3.77 (95% CI 3.68 to 3.85) in 1999 to 3.74 (95% CI 3.67 to 3.81) in 2020 (**Table 3**, **Figure 4**). In the urban regions from 1999 to 2020, there was a significant decrease in the APC and AAPC, of which both values were -0.16 (95% CI -0.29 to -0.01)* (**Figure 5**).

### Region and gender

**Figure 6:**
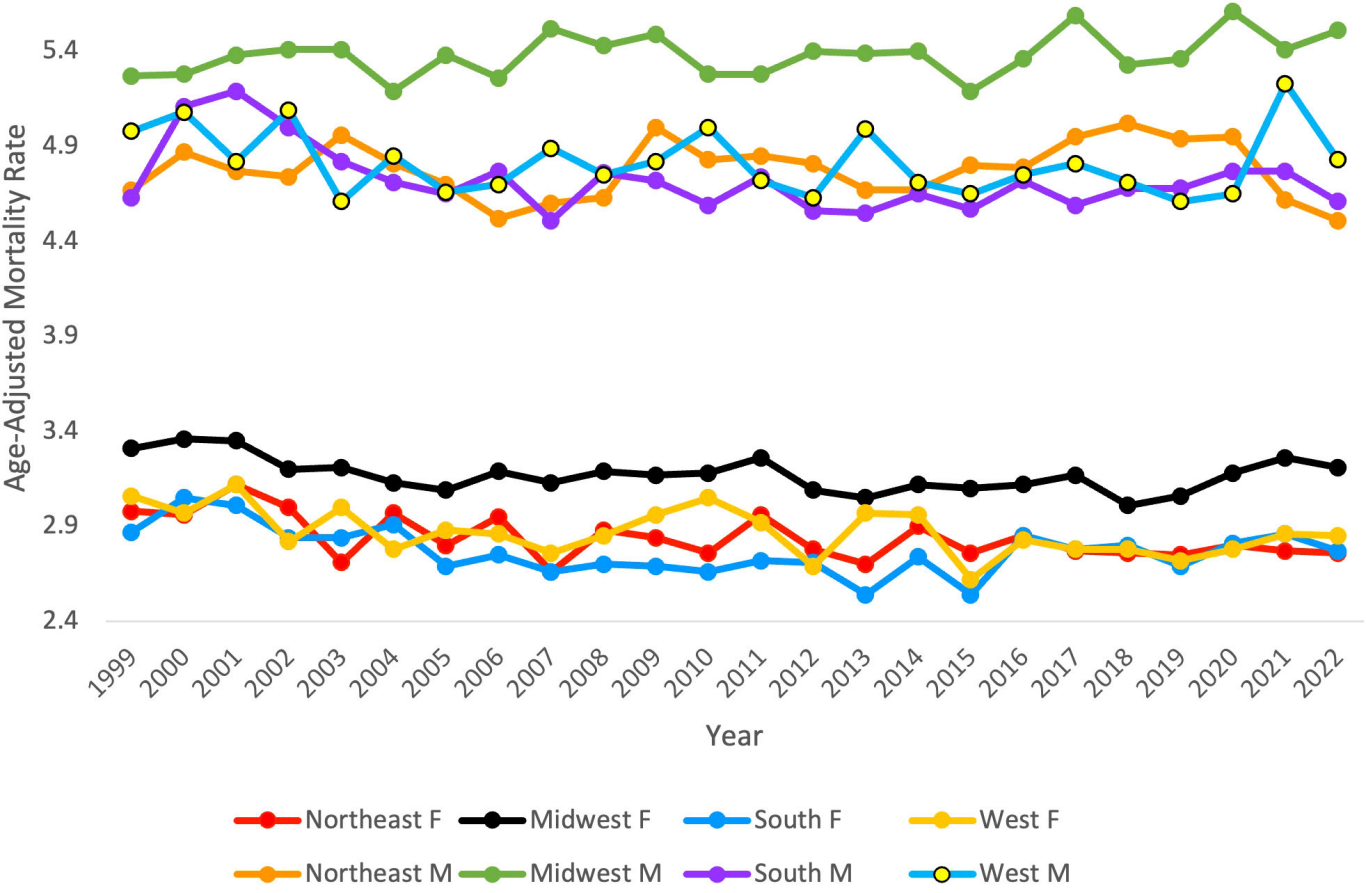
Myeloid Leukemia cancer age-adjusted mortality rate per 100,000 people; stratified by region and gender (F = Female, M = Male), 1999-2022.

**Figure 7:**
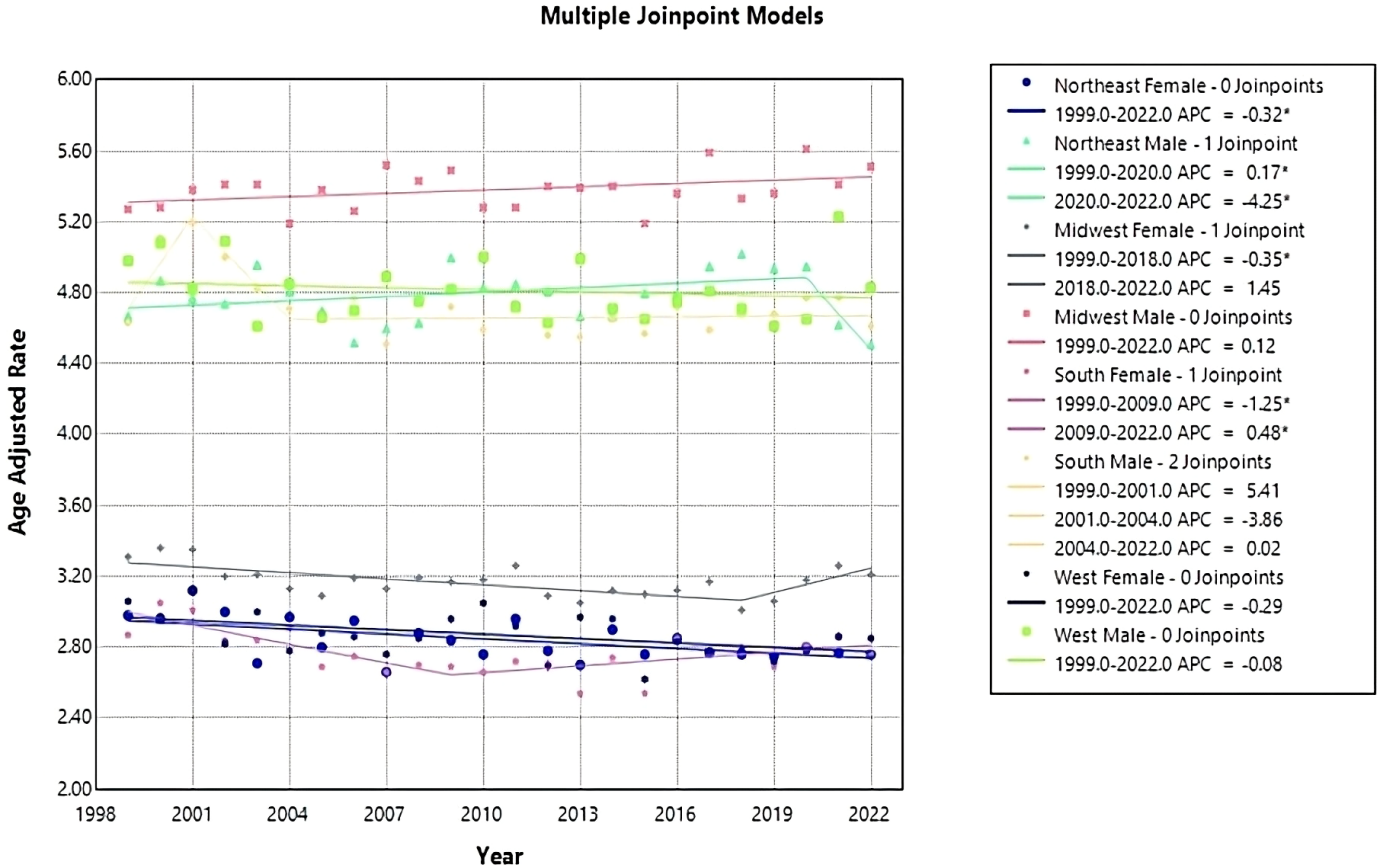
Joinpoint model of Myeloid Leukemia cancer-related AAMR per 100,000 people; stratified by region and gender, 1999-2022 (*indicates the APC is statistically significant).

From 1999 to 2022, ML resulted in 158,472 (56.73%) deaths in males and 120,883 (43.27%) in females in the United States. During this period, males consistently had the highest AAMR overall and across each census region (**Figure 6**). Males had the highest rate in 2020 in the Midwest, at 5.61 (95% CI 5.37 to 5.85) (**Figure 6**, **Table 4**). The lowest AAMR for males was in 2007 and 2022 in the South and Northeast, respectively, both at 4.51 (95% CI 4.32 to 4.71, South) and (95% CI 4.28 to 4.74, Northeast) (**Figure 6**, **Table 4**). In females, the highest AAMR was in 2000 in the Midwest, at 3.36 (95% CI 3.18 to 3.55) (**Figure 6**, **Table 4**). The lowest AAMR for females was in 2013 in the South, at 2.54 (95% CI 2.42 to 2.66) (**Figure 6**, **Table 4**). For males in the Northeast region, the APC in AAMR was significant at 0.17 (95% CI 0.01 to 0.84)* from 1999-2020, which then significantly decelerated to -4.25 (95% CI -7.22 to -0.18)* from 2020-2022 (**Figure 7**). Northeast females saw a significant decrease in APC of -0.32 (95% CI -0.53 to -0.11)* (**Figure 7**). Males in the Midwest did not see a significant difference in APC over time (**Figure 7**). Midwestern females had a significant decrease in APC of -0.35 (95% CI -1.20 to -0.11)* from 1999 to 2018 (**Figure 7**). Males in the South did not see a significant difference in APC over time (**Figure 7**). For females in the South, the APC from 1999 to 2009 significantly decreased at -1.25 (95% CI -2.98 to -0.56)*, which then significantly increased to 0.48 from 2009 to 2022 (95% CI 0.05 to 1.54)* (**Figure 7**). Neither males nor females in the West saw a significant APC over time (**Figure 7**).

**Table 4:** Myeloid Leukemia cancer age-adjusted mortality rate per 100,000 people stratified by region and gender, 1999-2022.

Year	Overall		Northeast		Midwest		South		West	
	Female	Male	Female	Male	Female	Male	Female	Male	Female	Male
1999	2.86	4.53	2.98	4.67	3.31	5.27	2.87	4.63	3.06	4.98
2000	2.92	4.80	2.96	4.87	3.36	5.28	3.05	5.11	2.97	5.08
2001	2.99	4.78	3.12	4.77	3.35	5.38	3.01	5.19	3.12	4.82
2002	2.79	4.77	3.00	4.74	3.20	5.41	2.84	5.00	2.82	5.09
2003	2.79	4.67	2.71	4.96	3.21	5.41	2.84	4.82	3.00	4.61
2004	2.80	4.58	2.97	4.81	3.13	5.19	2.91	4.71	2.78	4.85
2005	2.72	4.53	2.80	4.70	3.09	5.38	2.69	4.65	2.88	4.66
2006	2.77	4.54	2.95	4.52	3.19	5.26	2.75	4.77	2.86	4.70
2007	2.64	4.50	2.66	4.60	3.13	5.52	2.66	4.51	2.76	4.89
2008	2.72	4.59	2.88	4.63	3.19	5.43	2.70	4.76	2.85	4.75
2009	2.76	4.65	2.84	5.00	3.17	5.49	2.69	4.72	2.96	4.82
2010	2.71	4.58	2.76	4.83	3.18	5.28	2.66	4.59	3.05	5.00
2011	2.77	4.62	2.96	4.85	3.26	5.28	2.72	4.74	2.92	4.72
2012	2.68	4.53	2.78	4.81	3.09	5.40	2.71	4.56	2.69	4.63
2013	2.62	4.53	2.70	4.67	3.05	5.39	2.54	4.55	2.97	4.99
2014	2.74	4.49	2.90	4.67	3.12	5.40	2.74	4.65	2.96	4.71
2015	2.56	4.46	2.76	4.80	3.10	5.19	2.54	4.57	2.62	4.65
2016	2.76	4.51	2.85	4.79	3.12	5.36	2.85	4.72	2.83	4.75
2017	2.69	4.57	2.77	4.95	3.17	5.59	2.78	4.59	2.78	4.81
2018	2.63	4.53	2.76	5.02	3.01	5.33	2.80	4.68	2.78	4.71
2019	2.59	4.49	2.75	4.94	3.06	5.36	2.69	4.68	2.72	4.61
2020	2.62	4.41	2.80	4.95	3.18	5.61	2.81	4.77	2.78	4.65
2021	2.62	4.41	2.77	4.62	3.26	5.41	2.86	4.77	2.86	5.23
2022	2.63	4.28	2.76	4.51	3.21	5.51	2.77	4.61	2.85	4.83

### Region and race

From 1999 to 2022, White people consistently had the highest AAMR across each region, with the highest rate in 2017 in the Midwest, at 4.41 (95% CI 4.25 to 4.57) (**Table 5**). The lowest AAMR was in 2003 in the Northeast Asian or Pacific Islanders, at 1.22 (95% CI 0.75 to 1.86) (**Table 5**).

**Table 5:** Myeloid Leukemia cancer age-adjusted mortality rate per 100,000 people; stratified by region and race, 1999-2022.

Year	Northeast				Midwest				South				West			
	Asian	Black	White	Latino	Asian	Black	White	Latino	Asian	Black	White	Latino	Asian	Black	White	Latino
1999	2.24	3.27	3.73	2.91	3.17	3.30	4.15	2.66	2.10	3.08	3.74	2.83	3.06	4.01	4.03	2.73
2000	2.46	3.26	3.77	3.02	*	*	*	*	*	*	*	*	2.39	3.41	4.08	3.18
2001	2.21	2.89	3.91	3.02	2.74	3.32	4.22	1.54	2.42	3.26	4.12	3.03	2.94	3.38	4.15	2.65
2002	2.36	3.09	3.81	2.38	*	*	*	*	2.28	3.24	3.90	2.55	2.73	3.74	4.11	2.37
2003	1.22	2.48	3.82	2.42	*	*	*	*	1.77	3.28	3.83	2.65	2.52	3.97	3.98	2.48
2004	1.72	2.98	3.95	2.23	*	*	*	*	1.59	3.31	3.81	2.43	2.67	3.30	3.98	2.51
2005	2.44	2.82	3.68	2.61	*	*	*	*	1.79	2.80	3.71	2.49	2.42	3.22	3.96	2.61
2006	1.75	2.46	3.88	1.95	2.69	3.91	4.11	2.69	2.56	3.04	3.83	2.33	2.76	3.54	3.97	2.44
2007	2.89	2.84	3.59	2.46	2.03	3.34	4.27	2.33	1.99	2.91	3.67	2.05	2.70	2.83	3.99	2.45
2008	1.46	3.14	3.84	2.11	2.49	2.96	4.24	1.99	2.04	2.88	3.80	2.50	2.80	3.51	3.98	2.49
2009	2.05	2.40	4.00	3.15	2.34	3.19	4.30	2.58	2.37	3.06	3.73	2.48	3.15	3.50	4.11	2.5
2010	1.67	2.86	3.82	2.53	2.81	3.20	4.17	2.80	2.66	2.61	3.75	2.65	3.48	3.65	4.15	2.71
2011	2.37	2.95	3.94	2.39	3.29	3.09	4.25	1.79	1.60	3.08	3.79	2.32	3.33	3.42	4.02	2.49
2012	2.42	2.71	3.83	2.42	2.86	3.39	4.19	2.69	2.63	2.94	3.73	2.30	2.86	3.06	3.84	2.60
2013	1.84	2.75	3.77	2.36	2.08	3.73	4.12	2.99	1.64	2.75	3.68	2.44	3.02	4.24	4.14	2.83
2014	1.96	2.80	3.83	2.53	2.16	3.07	4.27	2.22	2.08	2.92	3.85	2.48	2.61	3.63	4.10	2.71
2015	2.47	2.71	3.86	2.30	1.99	3.13	4.11	2.43	2.21	2.88	3.66	2.22	2.70	2.55	3.89	2.49
2016	1.92	3.26	3.83	2.73	2.09	3.4	4.23	2.81	2.20	3.02	3.94	2.53	2.86	4.22	4.02	2.67
2017	2.45	3.22	3.90	2.22	1.91	3.14	4.41	2.21	2.32	2.94	3.88	2.55	2.96	3.65	3.98	2.67
2018	2.59	3.00	3.91	2.62	2.06	3.71	4.15	1.86	2.23	3.07	3.87	2.49	2.88	3.32	4.02	2.61
2019	2.26	2.57	3.95	2.61	2.61	3.20	4.18	2.23	2.63	3.30	3.76	2.60	2.80	3.46	3.91	2.53
2020	2.24	3.17	3.95	2.45	1.94	3.52	4.38	2.78	2.34	3.29	3.92	2.53	2.82	3.58	4.02	2.45
2021	2.27	2.68	3.84	2.42	2.20	3.60	4.38	2.44	2.27	3.00	4.10	2.47	2.86	4.00	4.35	2.76
2022	1.88	2.89	3.80	2.51	2.14	3.55	4.37	2.02	2.84	3.09	3.91	2.34	2.88	3.78	4.10	2.85

(*indicates unreliable data).

**Figure 8:**
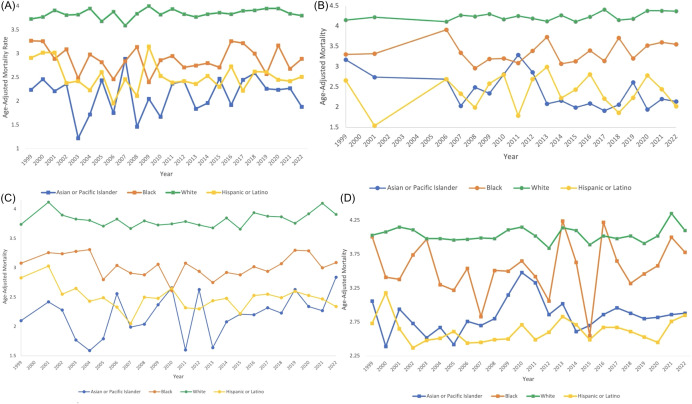
Myeloid Leukemia cancer age-adjusted mortality rate per 100,000 people; stratified by race, 1999-2022, in the **(A)** Northeast region, **(B)** Midwest region, **(C)** Southern region, and **(D)** Western region.

**Figure 9:**
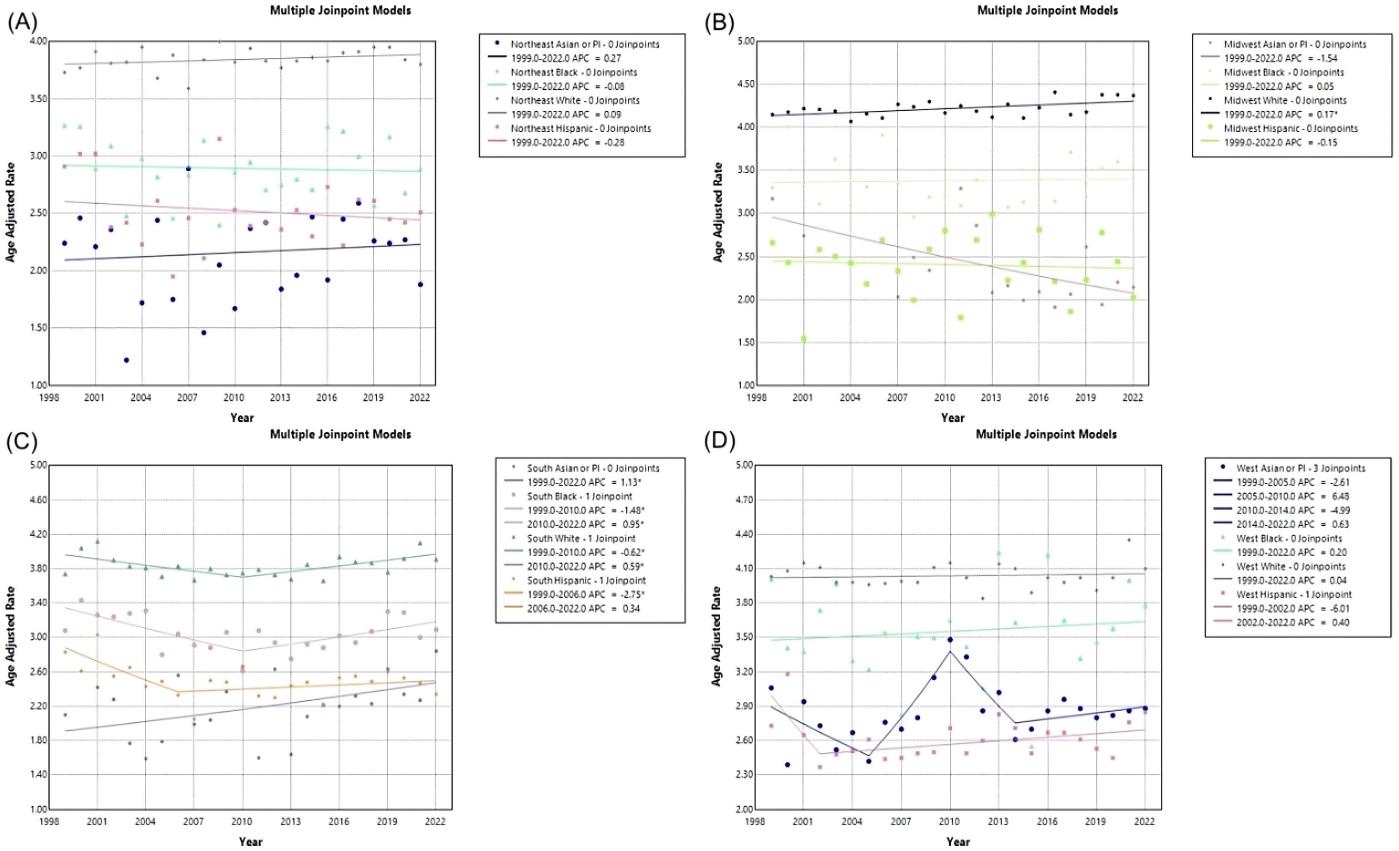
Joinpoint model of Myeloid Leukemia cancer-related AAMR per 100,000 people; stratified by race, 1999-2022, in the **(A)** Northeast region, **(B)** Midwest region, **(C)** Southern region, and **(D)** Western region. (*indicates the APC is statistically significant).

In the Northeast region, Asian or Pacific Islanders had the lowest AAMR, with 2.24 (95% CI 1.46 to 3.28) in 1999 to 1.88 (95% CI 1.48 to 2.35) in 2022, and a non-significant AAPC and APC of 0.271 (95% CI -0.77 to 1.70) (**Table 5**, **Figures 8**, **9**). In the Midwest region, the White race had a significant increase in APC at 0.17 (95% CI 0.03 to 0.31)* from 1999-2022 (**Table 5**, **Figure 9**). In the Midwest region, Asian or Pacific Islanders had the lowest AAMR, with 3.17 (95% CI 1.81 to 5.14) in 1999 to 2.14 (95% CI 1.54 to 2.89) in 2022, and a non-significant AAPC and APC of -1.54 (95% CI -2.80 to 0.01) (**Table 5**, **Figures 8**, **9**). In the Southern region, White people from 1999–2010 had a significant decrease in APC at -0.62 (95% CI -2.99 to -0.07)*, and from 2010-2022, they had a significant increase in APC at 0.59 (95% CI 0.14 to 2.59)* (**Figure 9**). Asian or Pacific Islanders had the lowest AAMR in the South, with 2.10 (95% CI 1.18 to 3.47) in 1999 to 2.84 (95% CI 2.34 to 3.34) in 2022, and a significant increase in AAPC and APC at 1.13 (95% CI 0.11 to 2.56)* (**Table 5**, **Figures 8**, **9**). Black and African Americans from 1999–2010 had a significant decrease in APC at -1.48 (95% CI -6.26 to -0.28)* and from 2010-2022, they had a significant increase in APC at 0.95 (95% CI 0.05 to 5.09)* (**Figure 9**). Hispanics and Latinos from 1999–2006 had a significant decrease in APC at -2.75 (95% CI -10.38 to -0.49)* and from 2006-2022, they had a non-significant APC of 0.34 (95% CI -0.21 to 2.87) (**Figure 9**). In the Western region, Hispanics or Latinos had the lowest AAMR, with 2.73 (95% CI 2.32 to 3.15) in 1999 and 2.85 (95% CI 2.58 to 3.12) in 2022 (**Table 5**, **Figure 8**). Hispanics or Latinos from 1999–2002 had a non-significant APC of -6.01 (95% CI -13.53 to 0.19) and from 2002–2022 had a non-significant APC of 0.40 (95% CI -0.23 to 2.77) (**Figure 9**). Asian or Pacific Islanders from 1999–2005 had a non-significant APC of -2.61 (95% CI -11.72 to 1.59), from 2005–2010 had a non-significant APC of 6.48 (95% CI -3.59 to 14.30), from 2010–2014 had a non-significant APC of -4.99 (95% CI -9.18 to 3.93), and from 2014–2022 had a non-significant APC of 0.63 (95% CI -1.02 to 6.36) (**Figure 9**).

## Discussion

From 1999–2007, ML-related mortality in the U.S. significantly declined, likely due to therapeutic advances such as the introduction of tyrosine kinase inhibitors for CML (3, 12). However, from 2007–2022, mortality showed a non-significant increase, despite newer therapies for AML like CPX-351 and Volasertib (3, 12). This plateau may reflect stagnation in treatment innovation for AML, which still relies heavily on the decades-old “3 + 7 regimen” (3, 12). The observed rise in mortality during recent years has been attributed in part to delayed diagnoses during the COVID-19 pandemic and growing resistance to therapies, particularly in older populations (6, 13).

Our analysis identified consistently higher AAMRs in the Midwest—a region that showed a non-significant upward mortality trend, in contrast to other U.S. regions. States like Iowa, Kansas, Minnesota, North Dakota, and South Dakota were among those with the highest mortality. Prior research has also highlighted this regional disparity, which may be linked to environmental exposures (e.g., agricultural chemicals), limited access to specialized care, and broader socioeconomic challenges (14–16). These structural factors may contribute to persistently high AAMRs, particularly in rural areas of the Midwest, where mortality has not improved over time.

Across all regions, rural areas experienced higher AAMRs than urban counterparts. Rural regions saw an initial decline in APC from 1999–2011, followed by a significant increase from 2011–2020. In contrast, urban areas experienced a sustained decline. These disparities may reflect differential access to healthcare resources, including proximity to cancer centers, availability of specialists, and socioeconomic conditions such as poverty and insurance coverage (16–19). Prior studies confirm that rural residence is independently associated with increased cancer mortality, even after adjusting for treatment and stage at diagnosis (20, 21).

Sex and race were also significant factors. Males had higher mortality rates than females in every region, with the highest rates in Midwestern males. This is consistent with previous literature (5, 14, 22). Similarly, the Midwest had the highest AAMRs for females. Racial disparities persisted: White individuals had the highest AAMRs, especially in the Midwest, while Asian or Pacific Islanders and Hispanic or Latino populations had the lowest. However, in the South, Asian or Pacific Islanders showed an increasing AAMR trend—highlighting a subgroup requiring further investigation.

The lack of consistent improvement in ML mortality, despite treatment advances, underscores systemic disparities. Enhanced access to novel therapies and specialized care—particularly in rural and underserved areas—should be a priority. In addition, culturally tailored interventions and regional public health strategies are needed to address worsening trends in specific demographic subgroups.

This study is limited by its reliance on aggregate mortality data from death certificates, which may be subject to misclassification and could obscure individual-level factors such as comorbidities or treatment adherence (23). Future studies should incorporate more granular, patient-level data to explore how environmental exposures, healthcare access, and socioeconomic conditions contribute to ML mortality across regions.

## Conclusion

This study provides a comprehensive evaluation of age-adjusted mortality rates (AAMRs) for myeloid leukemias (ML) across the United States from 1999 to 2022, identifying significant trends and disparities by region, state, urban/rural residence, gender, and race. While overall ML-related mortality rates have remained relatively stable, notable regional variations emerged, with the Midwest experiencing persistently higher AAMRs compared to other regions. Urban-rural disparities were evident, with rural areas consistently reporting higher mortality rates and concerning recent increases in APCs. Gender disparities also persisted, with males exhibiting higher mortality rates than females across all regions. Racial trends revealed that White individuals bore the highest burden, though some minority groups, such as Black and Asian or Pacific Islander populations, showed significant fluctuations in AAMRs over time.

The findings underscore the need for targeted public health interventions, improved healthcare access in rural and high-burden regions, and further research to address demographic disparities in ML mortality. Enhancing data sharing and leveraging large-scale databases like CDC WONDER will be instrumental in reducing ML-related mortality and driving evidence-based strategies to improve outcomes for affected populations.

## Data Availability

The datasets presented in this study can be found in online repositories. The names of the repository/repositories and accession number(s) can be found below: https://wonder.cdc.gov/ Centers for Disease Control and Prevention Wide-Ranging Online Data for Epidemiologic Research (CDC WONDER), Accession number: C92.
